# Retraction Note: MYC-mediated upregulation of PNO1 promotes glioma tumorigenesis by activating THBS1/FAK/Akt signaling

**DOI:** 10.1038/s41419-024-06847-8

**Published:** 2024-06-28

**Authors:** Xu Chen, Zheng-Qian Guo, Dan Cao, Yong Chen, Jian Chen

**Affiliations:** Department of Neurosurgery, Tongji Hospital, Tongji Medical College, Huazhong University of Science and Technology, Jiefang Ave, 1095, Wuhan, 430030 China

Retraction Note to: *Cell Death and Disease* 10.1038/s41419-021-03532-y, published online 04 March 2021

The authors have retracted this article after concerns were raised about the data. Specifically, there appear to be multiple different areas of overlap between the PNO1 panels in Figs. 5h and 6e. The authors no longer have confidence in the reliability of this article’s findings and conclusion.

All authors agree with this retraction.

